# Seasonal and environmental dynamics of intra-urban freshwater habitats and their influence on the abundance of *Bulinus* snail host of *Schistosoma haematobium* in the Tiko endemic focus, Mount Cameroon region

**DOI:** 10.1371/journal.pone.0292943

**Published:** 2023-10-19

**Authors:** Godlove Bunda Wepnje, Marcell K. Peters, Adeline Enjema Green, Tingmi Emparo Nkuizin, Daniel Brice Nkontcheu Kenko, Fairo F. Dzekashu, Helen Kuokuo Kimbi, Judith Kuoh Anchang-Kimbi

**Affiliations:** 1 Department of Animal Biology and Conservation, Faculty of Science, University of Buea, Buea, Cameroon; 2 Department of Animal Ecology and Tropical Biology, Biocenter, University of Würzburg, Würzburg, Germany; 3 International Centre of Insect Physiology and Ecology (*icipe*), Nairobi, Kenya; 4 Department of Biological Sciences, University of Lethbridge, Lethbridge, AB, Canada; 5 Department of Biomedical Sciences, Faculty of Health Sciences, University of Bamenda, Bambili, Cameroon; 6 Department of Microbiology and Immunology, Drexel University College of Medicine, Philadelphia, PA, United States of America; UNIMED: University of Medical Sciences Ondo City, NIGERIA

## Abstract

Urogenital schistosomiasis (UGS) caused by *Schistosoma haematobium* is endemic in the South West Region of Cameroon. An understanding of the abundance and distribution of the *Bulinus* snail, intermediate host can inform strategic snail control programmes at a local scale. This study investigated seasonal dynamics and environmental factors influencing occurrence and abundance of freshwater snail intermediate hosts in Tiko, a semi-urban endemic focus in the Mount Cameroon area. A longitudinal malacological field survey was conducted between December 2019 and December 2020 in the Tiko municipality. Snails were collected for one year monthly at 12 different human water contact sites along a stretch of the Ndongo stream using a standardized sampling technique. Freshwater snails were identified using shell morphological features. In addition, water temperature, pH, electrical conductivity, total dissolved solutes, salinity, water depth, width and flow velocity were measured, and vegetation cover as well as substrate type were determined. Bayesian regression models were used to identify the main environmental factors affecting the occurrence and abundance of *Bulinus* intermediate host. In total, 2129 fresh water snails were collected during the study period. *Physa* (51.4%) was the most abundant genus followed by *Melanoides* (28.6%) then, *Bulinus* (15.5%), *Lymnaea* (4.2%), *Indoplanorbis* (0.2%) and *Potadoma* (0.1%). Seasonality in abundance was significant in *Bulinus* sp as well as other genera, with greater numbers in the dry season (peaks between December and February). Water temperature, a rocky or sandy substrate type associated positively with *Bulinus* sp, meanwhile a higher water flow rate and medium vegetation negatively influenced the snail intermediate host population. These findings underscore the importance of timing behavioural and snail control interventions against schistosomiasis as well as increase vigilance of other trematode diseases in the study area. The continuous spread of planorbid snail hosts is a major concern.

## Introduction

Schistosomiasis is a parasitic helminthic and widespread neglected tropical disease in many developing countries [1]. Transmission of schistosome parasites does not occur directly from person to person, but requires freshwater where the snail intermediate hosts live and breed. Snail hosts are critical for the development, multiplication, and transmission of schistosomes implying that both the parasite and the intermediate host must be targeted in order to break the cycle of transmission so as to achieve effective control against the disease [2]. *Biomphalaria*, *Bulinus*, and *Oncomelania* snail species are responsible for larval development for the human *Schistosoma* parasite species namely, *S*. *mansoni*, *S*. *haematobium*, and *S*. *japonicum*, respectively [3, 4]. *Schistosoma haematobium* is the most widespread schistosome species across Africa with risk of infection in freshwater in southern and sub-Saharan Africa including the great lakes and rivers as well as smaller bodies of water [5, 6].

The focal distribution of schistosomiasis reflects to a large extent the distribution of the genera and species of intermediate snail hosts that are compatible with the parasite [7–9]. Also, lack of adequate sanitation and clean water and, more importantly, human water contact behaviour play a role in the transmission of schistosomiasis through the release of eggs in the environment by infected people and contact with contaminated water. When *Schistosoma* eggs are eliminated into freshwater bodies with urine or faeces, depending on the species, eggs hatch and release miracidia which penetrate specific snail intermediate hosts. The stages in the snail include two generations of sporocysts and the production of cercariae. Upon release from the snail, the infective cercariae swim, penetrate the skin of the human host and becomes schistosomulae [10, 11]. After entering the definitive host, the schistosome larvae mature into adult worms in the blood vessels of the liver, intestine and bladder. *Schistosoma haematobium* adult worms reside and copulate in vesicular and pelvic venous plexus of the bladder [10, 11]. The worms lay thousands of eggs which are lodged within organs of the urogenital tract eliciting inflammatory reactions, scaring and consequent progressive damage to organs [12]. To curb morbidity and eradication of schistosomiasis by 2030, WHO recommends extending preventive chemotherapy to all populations in need; targeted snail control and continued micro-mapping as critical effective interventions [13]. Integration of snail control as a preventive measure requires a thorough understanding of snail distribution.

The snails inhabit a wide range of habitats because they are found in various freshwater environment and ecological niches such as ponds, ditches and other humid areas consisting of open water, aquatic vegetation and/or inundated grass [14, 15]. Environmental and climatic factors have been well studied in regards to how they affect the population size and density of the intermediate host snails for schistosomiasis [16–24]. Environmental factors include physical and chemical water properties such as temperature, turbidity, salinity, conductivity, pH and velocity and biological factors such as the availability of food, competition, predator-prey interactions, presence, and density of aquatic plants. Limiting factors for snail habitat preferences are well established. Rainfall and temperature have been reported as the main climatic factors that determine fecundity and mortality of planorbid freshwater snails [16, 24–28]. Reports have indicated that human activities, like waste disposal, are strongly associated with the presence of snail species in heavily disturbed areas, primarily due to the abundance of organic matter and dissolved ions that are beneficial to the snails [29]. Recently, agricultural development and associated fertilizer use in West Africa are increasing the burden of schistosomiasis by fostering the growth of aquatic plants that serves as habitat for freshwater snails that transmit schistosome [30]. Nevertheless, the importance of these factors can vary considerably from one ecological zone to another and even from one aquatic habitat to another in the same ecological zone [23, 31, 32]. Thus, it is important to identify factors at a micro-geographic level that influence snail habitat preference for better understanding of local transmission dynamics.

In Cameroon, urogenital schistosomiasis is prevalent in Northern, Western and Southwestern regions of Cameroon [33, 34] where transmission of the disease is maintained by the presence of *Bulinus* sp [35]. Although *Bulinus* sp snail populations have been characterized in several endemic foci in Western Region [36], Southwest Region [37] and Centre Region [38], there exist limited studies on the ecology and population dynamics of intermediate host freshwater snails in Cameroon [37, 39]. Urogenital schistosomiasis surveys have established variation in the occurrence of the disease ranging from 12% to 57% among affected communities in Tiko, a semi-urban endemic focus situated at low altitude in the Mount Cameroon Area [40, 41]. To elucidate the epidemiology of *S*. *haematobium* infection in the Tiko focus, we conducted a malacological survey to identify and determine seasonal and population dynamics of *Bulinus* sp in human contact intra-urban water habitats in the Tiko endemic focus by assessing abiotic and biotic factors influencing these patterns. This study can provide useful data for development and optimization of available control interventions in the affected communities.

## Methods

### Study area and location of sampling sites

**Fig 1:**
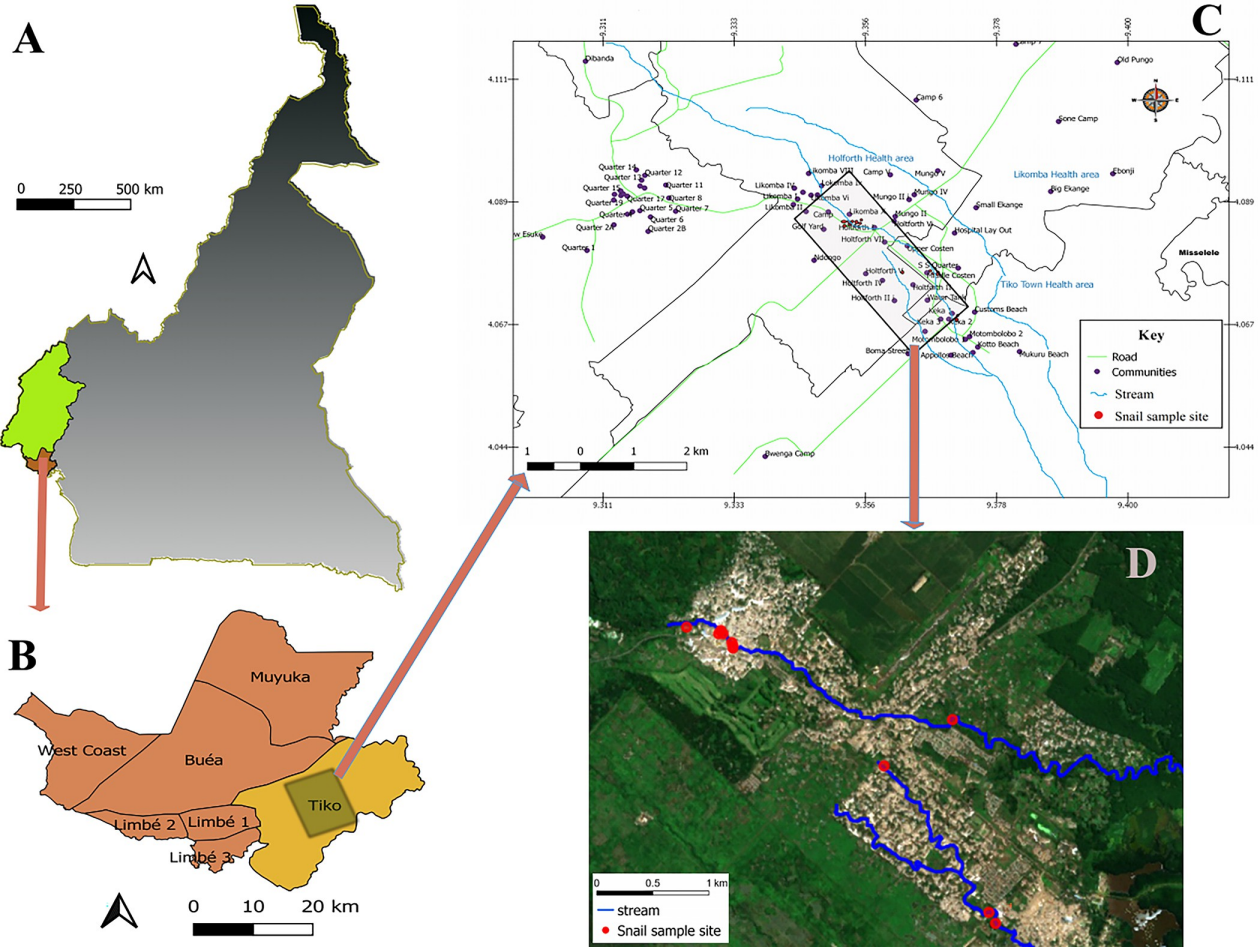
Map of the study region and location of the water contact sites. (A) Topographic map of Cameroon (grey) with the South West region (highlighted green) and Fako division (oxblood color). (B) Map of Fako division with Tiko subdivision (highlighted yellow) and the study area (grey square). (C) Map of the study sites within Tiko subdivision. The black box indicates the focal sampling area. (D) Aerial photograph of Tiko municipality showing the location of the 12 water contact sampling sites (red dots). Image (D) of the study area was derived using Sentinel 2 true colour RGB imagery, source: Copernicus Sentinel data, 2023.

This malacological study is part of a larger epidemiological study carried out in the Tiko Health District [41]. We conducted field sampling for twelve consecutive months (i.e., from December 2019 to December 2020) at several human-water contact sites (hereafter referred to as ‘sites’) along a stretch of the Ndongo stream from Likomba SNEC area (4°09´ N to 9°34´E, 92 m asl) to Ndongo water tank (4°07´ N to 9°37´E, 21 m asl) in the coastal plains of Tiko Municipality, Tiko Sub-division, South West Region, Cameroon (Fig 1). The climatic conditions of the area are warm and humid, typical of coastal equatorial tropical climate, but differ from that of the central and northern parts of the country [42]. Agriculture is the main source of livelihoods for the local indigenes. They either work in small scale subsistence farms and/or in large scale banana, oil palm and rubber plantations of the Cameroon Development Corporation (CDC).

This coastal area is characterized by two conspicuously distinct seasons, the rainy and dry seasons. Here, the rainy season occurs from April to November and the mean annual precipitation ranges from 2000 mm to 5000 mm and is closely followed by a warm dry period of four months that lasts from December to March [42]. The mean daily temperatures are relatively constant throughout the year and ranges between 28°C and 33°C in the rainy and dry seasons respectively [43]. The open water sources in the Tiko municipality include; River Moungo and Ombe, Ndongo and Benyo streams [41]. Due to inadequate water supply in the area, these water sources are used by the indigenes for domestic (bathing, washing and other kitchen activities), economic (washing of motorcycles) and recreational (swimming) purposes [40, 41].

### Snail survey

Three health areas (i.e., Holforth, Likomba and Mutengene; Fig 1) were purposively selected for the survey based on the presence of human water contact points. A total of 12 sites (i.e., streams and springs) were identified across the health areas considering accessibility to anthropogenic activities and existing variations in ecological characteristics of the habitats. The “Ndongo” stream flows across the different communities namely Likomba (NL), Consten (NC), Holforth (NH) and Water tank (NWT). Also, one spring (SNEC) was selected for sampling. Before site visits, an authorization was obtained from the local authorities (chiefs/quarter heads) of the various communities before sampling any water contact point. Sampling sites were fixed at ~ 5m^2^, h whereas length of 10m along the stream shoreline was sampled. Snail host sampling surveys were conducted consecutively for a full year covering the two seasons of this area. All sites were georeferenced using a hand-held global positioning system (GPS; Garmin Sery GPS MAP 62, Olathe, KS, USA) device.

Sampling was standardised for each site and snails were collected by two experienced observers by either hand picking using synthetic gloves or using locally made long-handled water nets (1m long wooden handle frame with mesh size of 2 × 2 mm) for a period of 30 minutes [2] once a month for a full year. We made certain of equal sampling possibilities by solely conducting sampling events during rain-free and or light rainy periods. Snails collected from infested streams were placed in plastic containers containing a sample of water and few micro plants from the water-contact site before onward transfer to the Zoology Research Laboratory of the University of Buea for identification using shell morphology and cercarial release by the shedding method.

### Snail identification and relative abundance

Identification of snail specimens to the level of genera was carried out by observing the shell morphological characteristics using standardised taxonomic identification keys [44]. Snails with globose, ovate and sinistral shells with blunt apex were identified as *Bulinus*. *Bulinus* were further identified as *Bulinus camerunensis* (longer aperture more than half the total shell length and shell slightly broader) and *Bulinus truncatus* (prominent spire and whorls somewhat shouldered) [45, 46].

After identification, potential intermediate hosts of schistosome were examined for infections status under a dissecting microscope. Individual snail was placed in a petri dish containing 1ml of dechlorinated tap water and exposed to artificial light (fluorescent bulb 75 WAT) for 4 h (between 10:00 am and 2:00 pm) to stimulate the release of cercariae. Snail exposure to light was repeated the next day to confirm status of cercariae shedding. All identified snails were counted and preserved in well labelled plastic Eppendorf tubes (2mL) containing 70% alcohol.

### Assessment of water physicochemical and hydrological properties

Water conductivity, total dissolved solids, salinity and temperature were quantified at each site using portable electronic multipurpose meter (Extech Instrument Corp. Waltham, USA) and pH meter (Hanna Instruments inc, Leighton Buzzard, Bedfordshire, UK).

Hydrological variables (velocity and discharge) were determined using the floating technique as described by Barbour et al. [47]. Flow velocity was obtained using a 1cm^2^ floating polystyrene. This object was placed at a distance of ~10 m upstream and marked as point zero. The time was recorded using a stop watch to the nearest second, when the floating polystyrene passed the zero point and the end point (10 meters). The rate of water velocity was calculated as shown in Eq 1 below.

$$Velocity\;(m/s)=\frac{\mathrm{Distance}\;(m)}{\mathrm{Time}\;(s)}$$ Eq (1)

The rate of water discharge was defined as the product of water width, depth and velocity (Eq 2). As such, water depth and width were measured for each sampling site using a measuring tape (mark).


$$Discharge\;(m^{3}/s)=Width\;(m)\;x\;Depth(m)\;x\;Velocity\;(m/s)$$ Eq (2)

### Assessment of vegetation and soil properties

Classification of vegetation cover was visually performed by an estimation of the proportion of sites covered by aquatic plants around a 20m radius from the centre of individual sampling sites [23]. The percentage cover of vegetation by aquatic plant species was classified into three categories as follows: Light vegetation (0–30% submerged soft-stemmed vegetation), medium (30–60% partially submerged vegetation) and heavy vegetation (60–100% fully emerged vegetation) [48]. Furthermore, at each sampling site, the soil type was equally classified into three categories namely: rocky, sandy and muddy soils as observed in the different sampled water bodies.

### Ethical statement

The study was approved by the Institutional Review Board hosted by the Faculty of Health Sciences, University of Buea (2019/922‑01/UB/SG/IRB/FHS) taking into consideration administrative clearance granted by the Southwest Regional Delegation of Public Health. Also, authorizations to permit access to field sites were obtained from the District Medical Officer for the Tiko Health District and the Community leaders who gave their consent after reading through the information sheet.

### Data analysis

All statistical analyses were conducted within the R statistics platform version 4.0.3 (R Development Core Team 2020) using the following packages: ‘brms’ (Bayesian Regression Models using Stan) [49], ‘corrplot’ ‘nlme’ (linear and nonlinear mixed effect models) [50] and ‘ggplot2’.

We calculated correlation coefficients of pairwise Pearson correlations among the major water flow, water quality parameters and snail species abundance and visualized them using the R package ‘corrplot’.

Upon observing high seasonal patterns in snail abundance, particularly of the main snail host responsible for *Schistosoma* infection (*Bulinus sp*.), we assessed which factors drive changes in the seasonal (temporal) distribution of the *Bulinus sp*. and analysed the influence of water flow continuous predictor variables (T°, TDS, EC, pH, Salinity and Flow rate) and factorial predictor variables (vegetation and soil type) by fitting Bayesian generalised linear mixed effect models with Stan (R package ‘brms’: Bürkner, 2022). Stan implements Hamiltonian Monte Carlo and its extension, the No-U-Turn Sampler. As multiple water flow measurements (flow velocity, flow rate, water width) turned out to be strongly correlated we only included the flow rate in the analysis. For modelling, the Poisson data family was used and the model included a temporal autocorrelation term and site was set as a random factor to account for correlated observations throughout the study period and location. The model was run with four independent Markov chains of 2000 iterations, using weakly informative priors (default settings) and discarding the first 1000 iterations per chain as warm-up, which resulted in 4000 posterior samples overall. Convergence of the four chains and sufficient sampling of posterior distributions were confirmed by the visual inspection of parameter traces and by ensuring a scale reduction factor r of 1.00. For each model, mean and 95% credible intervals were calculated from posterior samples. Credible intervals of all continuous predictor variables were standardized using the *scale* function, to allow a direct comparison of effect strengths among predictor variables.

## Results

### Occurrence of freshwater snails

**Fig 2:**
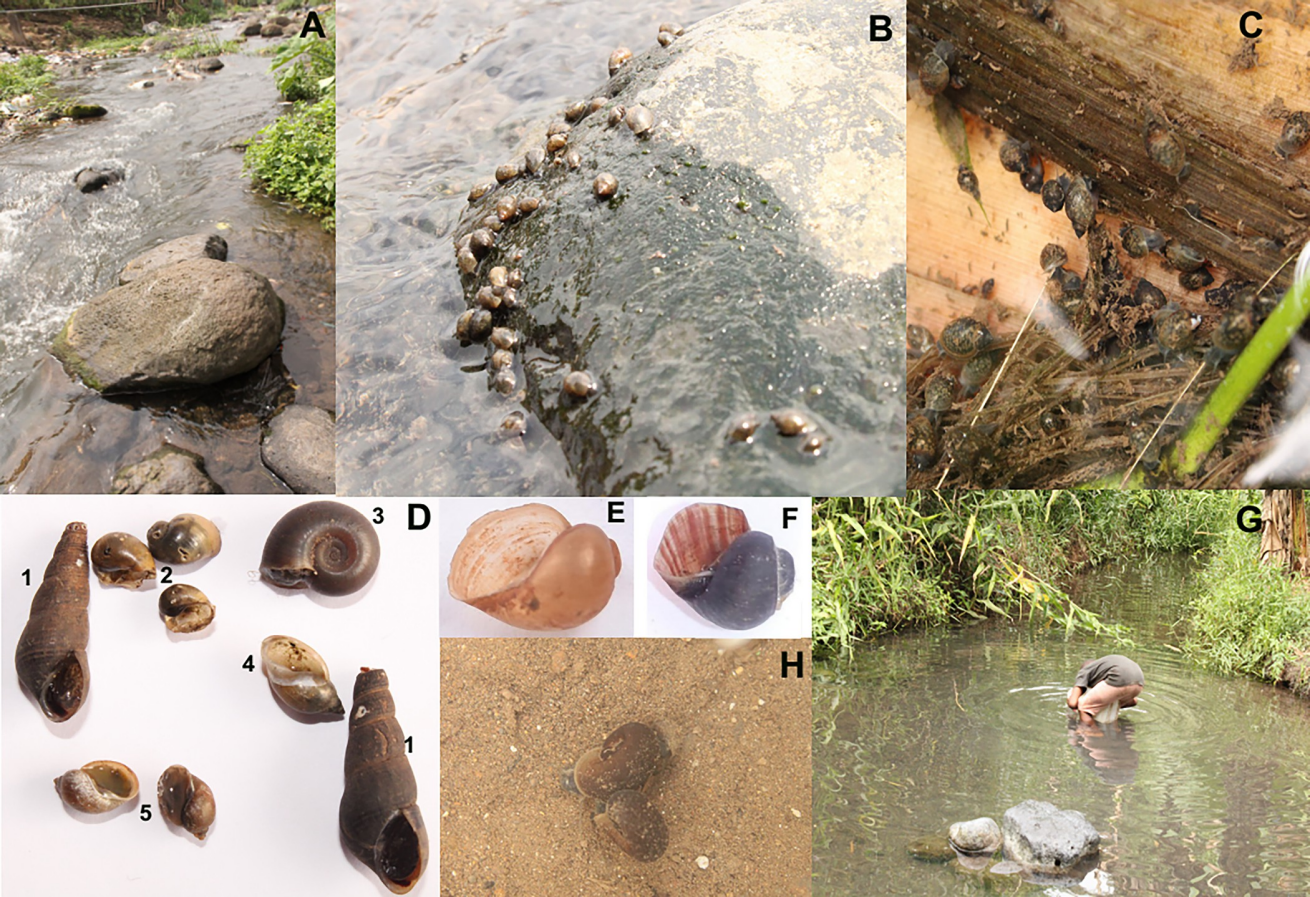
Photographs of study sites, snail specimens and human activities on study sites. Photograph of a free-flowing freshwater contact site (A). Photographs of snail communities on a rocky surface along water bodies (B). Snail assemblages on a plantain stem submerged in a shallow stream (C). Photographs of some snails collected during sampling events (D), *Melanoides sp*. (D1), *Bulinus sp*. (D2), *Indoplanorbis sp*. (D3), *Physa sp*. (D4) and *Lymneae sp*. (D5). Detailed photo of the snail host *Bulinus camerunensis* (E) and *Bulinus truncatus* (F) responsible for *Schistosoma* infection. A photo of human contact activity (here, a man fetching water for domestic purposes) (G). Photo of *Indoplanorbis sp*. inside a free-flowing fresh water (H).

A total of 2129 individual snails were putatively identified belonging to five families and six genera during the entire sampling survey (Fig 2). The most abundant family was Physidae (1094 individuals) followed by the families Thiaridae (608 individuals), Planorbidae (335 individuals), Lymnaeidae (89 individuals) and Pachychilidae (3 individuals). *Bulinus* which is an intermediate host for *Schistosoma*. *haematobium* made up 15.5% (331) of the total number of snails collected. On the basis of shell morphology, further identification of the genus *Bulinus* led to the identification of two species i.e. *Bulinus truncatus* and *Bulinus camerunensis*. An invasive genus, *Indoplanorbis* spp of the subfamily Bulininae was also identified (1.2%; 4).

The most abundant genus was *Physa* (n = 1094, 51.4%), followed by *Melanoides* (n = 608, 28.6%) then, *Bulinus* (n = 331, 15.5%), *Lymnaea* (n = 89, 4.2%), *Indoplanorbis* (n = 4, 0.2%) and *Potadoma* (n = 3, 0.1%). Interestingly, with respect to different sites, *Bulinus* was conspicuously absent from site 10 throughout the different sampling rounds. Also, *Indoplanorbis* was only reported in sites 11 and 12 as shown in Table 1.

**Table 1:** Distribution of different freshwater snails sampled per study site and summed across the different sampling periods in Tiko.

Site	Site location*	*Physa sp*	*Bulinus sp*	*Melanoides sp*	*Lymneaea sp*	*Potadoma sp*	*Indoplanorbis sp*
S1	NL-SN	133	7	79	27	3	0
S2	NL-1	201	17	24	3	0	0
S3	NL-2	57	39	16	8	0	0
S4	NL-3	164	30	43	3	0	0
S5	NL-4	68	33	39	1	0	0
S6	NL-5	72	91	144	8	0	0
S7	NL-6	41	19	23	7	0	0
S8	NL-7	117	43	2	5	0	0
S9	NC	93	12	109	19	0	0
S10	NH	24	0	116	1	0	0
S11	NWT-1	39	2	3	1	0	2
S12	NWT-2	85	38	10	6	0	2
**Total**		**1094**	**331**	**608**	**89**	**3**	**4**

*Sites purposively selected during human contact activities and included for snail sampling

• SN = SNEC

• NL = Ndongo-Likomba

• NC = Ndongo-Costen

• NH = Ndongo-Holforth

• NWT = Ndongo-Water tank

### Occurrence of seasonal snail species abundance

**Fig 3:**
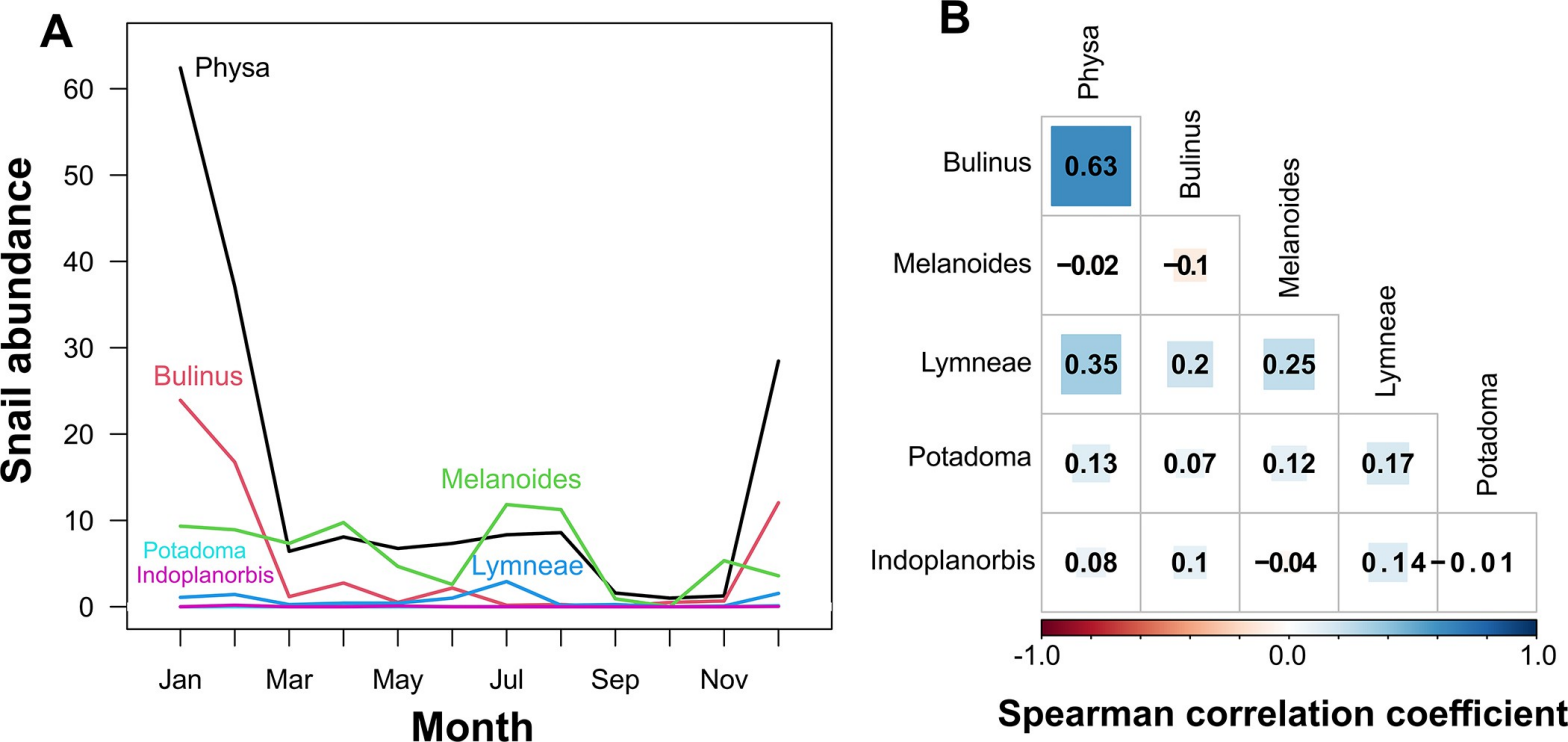
Seasonal change in the mean abundance of *Bulinus* snails and of other aquatic snail taxa (averaged across rivers). A) Abundance of snails of the two abundant genera *Bulinus* and *Physa* peaked in between December and February and were very low between March and November. B) Correlation matrix showing coefficients of pairwise Spearman correlations of snail abundances.

There was a marked shift in abundance of the most abundant genera (*Physa*) and the main snail intermediate host responsible for *S*. *haematobium* infection (*Bulinus sp*.) over the year and across seasons (Fig 3A), such that abundance significantly increased during the dry season (with peaks in between December and February) than in the rainy season (very low between March and November) for *Physa* (mean±sd dry = 22±5, mean±sd rainy = 6±1, F = 17.7, p < 0.001, Fig 3A) and *Bulinus* (mean±sd dry = 9±2, mean±sd rainy = 1±0, F = 20.7, p < 0.001, Fig 3A) respectively. Moreover, *Bulinus* and *Physa* abundances were strongly positively correlated while all other taxa showed idiosyncratic changes in abundance over the year (Fig 3B).

**Fig 4:**
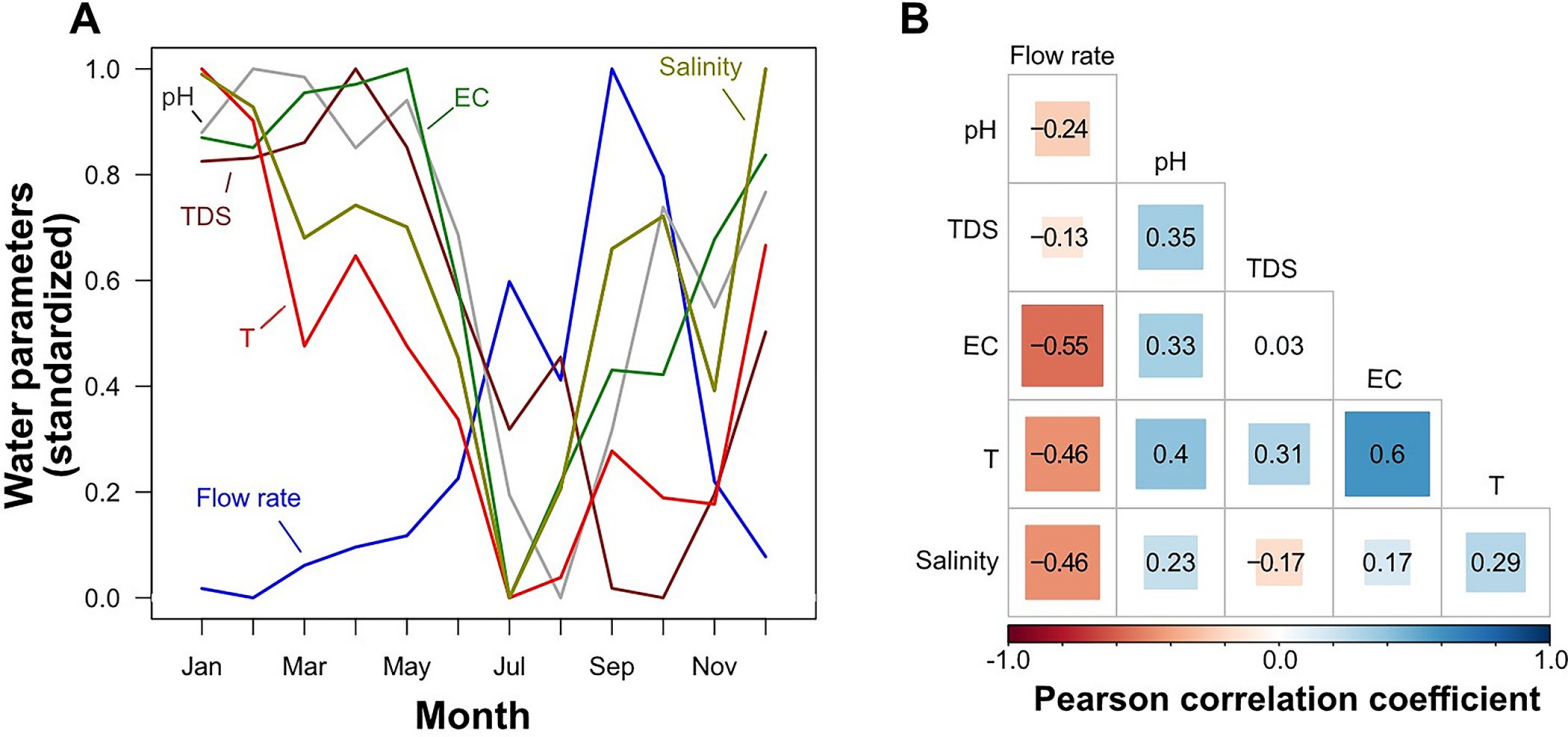
Seasonal changes in mean (averaged across rivers) water parameters over a one-year period (A). Correlation coefficients of pairwise Pearson correlations of water parameters (B).

Flow rate of water (originally measured in m^2^ per sec) increased from January to September and then decreased to lowest values in December/January (Fig 4A). Water temperature (T), pH, electrical conductivity (EC), total dissolved solids (TDS) and salinity peaked in January and decreased to lowest values between July and October to increase to highest values in December/January (Fig 4A). Moreover, high water flow was negatively correlated with EC, T, Salinity, and to a lesser degree with pH and TDS. Water temperature and EC were positively correlated (Fig 4B).

### Drivers of *Bulinus*spp abundance across seasons

**Fig 5:**
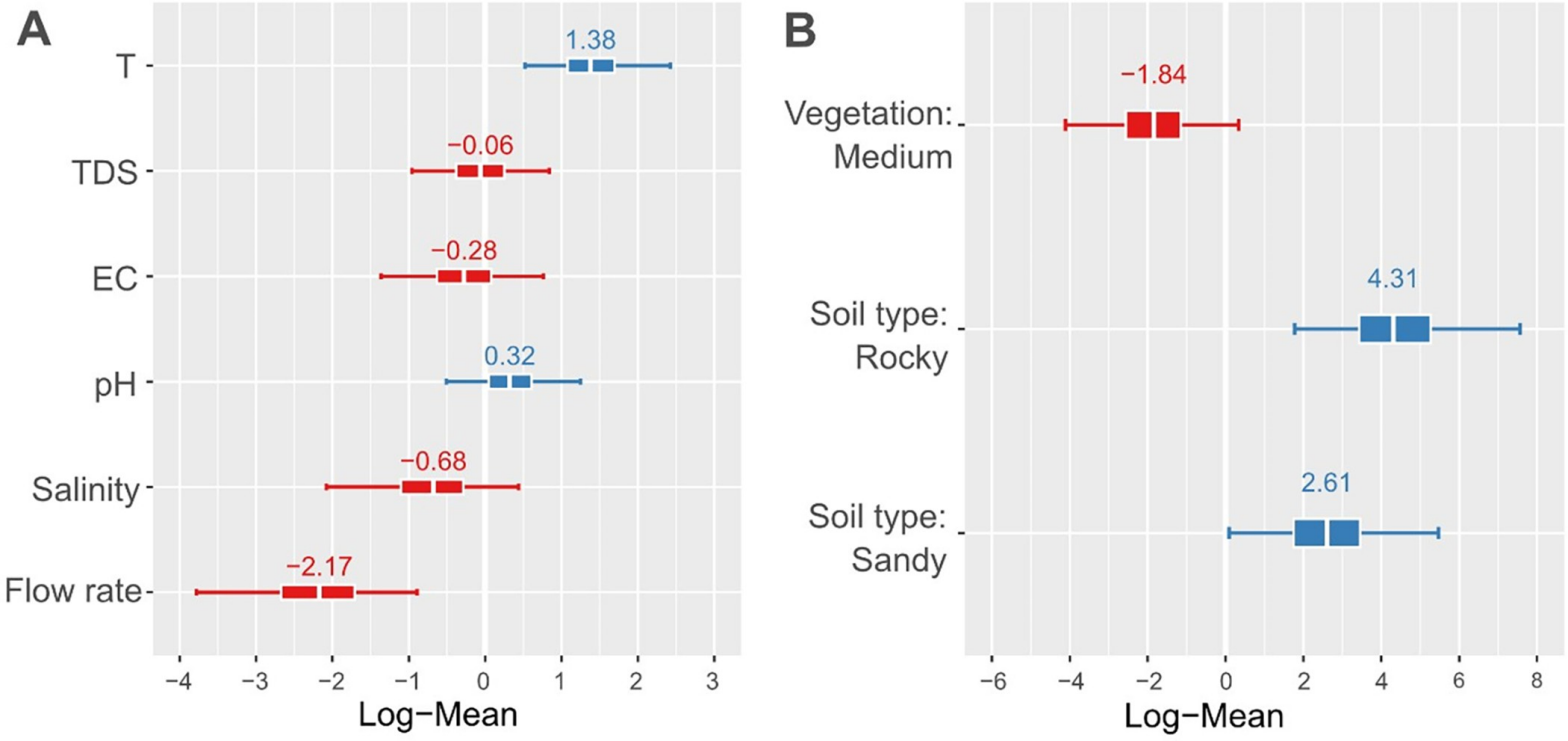
Statistical support for water/stream variables influencing the *Bulinus* abundance. Shown are mean and 95% credible intervals (whiskers) of parameter estimates for predictor variables of *Bulinus* abundance from a Bayesian hierarchical model (with a temporal autocorrelation term and stream as a random factor). Mean parameter estimates are shown by a white line in the center of each box and by values above each box-and-whisker plot. Boxes give 50% quantiles of estimates. Positive and negative effects are indicated by blue and red box-and-whisker plots respectively. A) Credible intervals of continuous predictor variables (scaled before analysis). B) Credible intervals for factorial predictor variables given as contrasts to the level which is not shown (for Vegetation: low; for Soil type: Muddy) T: Temperature, TDS: Total Dissolve solids, EC: Electrical conductivity).

Our multivariate analyses using Bayesian regression models revealed that *Bulinus* snail abundance was shaped by several factors. We found strong support for positive effects of temperature and negative effects of flow rate on the abundance of *Bulinus* spp (95% credible intervals not overlapping zero) as shown in Fig 5. Furthermore, *Bulinus* spp was found to be more abundant on rocky and sandy than on muddy substrates (95% credible intervals not overlapping zero). Additionally, a negative effect of vegetation cover was revealed (with 95% CI slightly overlapping with zero). In contrast, EC, TDS, salinity nor pH did not affect *Bulinus* abundance (all 95% CI overlapping zero).

**Fig 6:**
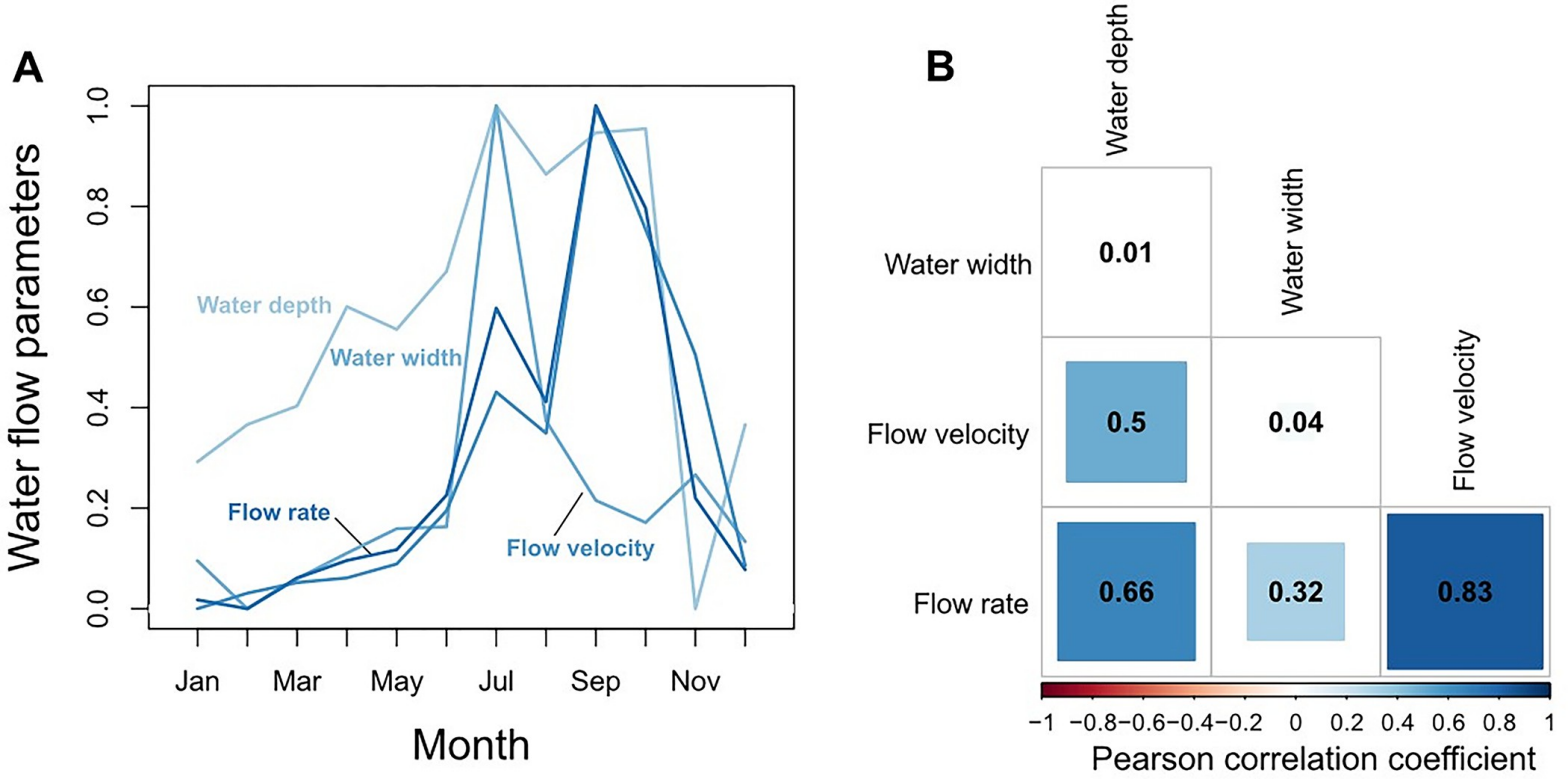
A) Seasonal change in water flow parameters (averaged across rivers) over a one-year period. Please note that for reasons of better comparability all water parameters were standardized by their range. B) Flow rate is moderately to strongly correlated to water width, water depth and flow velocity.

Water flow parameters showed significant variations within the study period (Fig 6A). Flow rate correlated with water width, water depth and flow velocity as shown in Fig 6B.

## Discussion

The ecology and population dynamics of snail intermediate hosts at a micro-geographical level are fundamental to the understanding of local transmission dynamics to plan and implement effective snail borne diseases’ prevention and control strategies [51]. Urogenital schistosomiasis is endemic in Tiko, Mount Cameroon area [41, 52]. Until now studies on UGS carried out in this area focused only on parasitological aspects (e.g., prevalence of infection), meanwhile none was directed to the transmission sites and snail host species involved. It is well known that mapping prevalences does not necessarily indicate the origin of infections, particularly in urban areas, where the mobility of the population is high [53]. This study findings demonstrate that the intra-urban fresh water bodies in this endemic focus provide suitable habitats for *Bulinus*, which is of great medical importance since it provides all the known snail host species for *S*. *haematobium*. Eleven out of the 12 sampled sites showed occurrence of *Bulinus* sp indicating that these frequently visited human water contact points are potential transmission hotspots for UGS. Also, this study revealed that seasonal changes in the abundance of freshwater snail species, and water temperature, flow rate and substrate type as the most important drivers of *Bulinus* snail abundance in the Tiko focus.

Six different freshwater snail genera were identified with each site having at least three or more different snail genera, an indication of snail coexistence in the same ecosystem. This agrees with the findings by Manyangadze et al. [54] who observed evidence of cohabitation between *Bulinus* and other freshwater snails. Among the freshwater snails identified, only two genera *Bulinus* and *Lymnaea* are known to serve as obligate intermediate hosts in the transmission of schistosomiasis and fascioliasis respectively in the subtropical regions of the world. Other freshwater snail genera such as *Physa*, *Melanoides and Potodama* with no prominent medical or veterinary importance have received less attention [55]. Two species of *Bulinus* were identified upon shell morphology including *B*. *truncatus* and *B camerunenesis* [44]. *Bulinus camerunenesis*, known only from crater lakes and proved to transmit *S*. *haematobium* in Lake Barombi Kotto [37] whereas *B*. *truncatus* is more or less widespread inhabiting small streams, seasonal pools, irrigation systems, dams, and lakes. Similarly, the two species of *Bulinus* and *Indoplanorbis* spp (Sub family Bulininae), an invasive southern Asian species recorded in Tiko were previously identified in lakes Barombi Kotto and Barombi Mbo endemic foci in Kumba, South West Region of Cameroon [37]. These planorbid species are known to be transported by man through aquatic plant trade or pisciculture and establishing viable populations in new habitats and creating new transmission foci [56]. During our interactions in different communities, we observed among the residents ignorance of the health risks associated with the snail intermediate hosts and inability to identity freshwater snail types within the environment. Hence, there is a need to educate and enlighten the community on disease-borne snail hosts as well as, increase vigilance for other trematode diseases.

There was a significant effect of seasonality on the abundance of all freshwater snails surveyed, with higher numbers found in the dry season, and reductions in the rainy season. These dynamics of snail populations is associated with abiotic factors, likely working in concert; for instance, snail displacement in wet months as water depth rises, and flow rate increases thereby limiting snail abundance. A negative association among snail distribution, abundance and rainfall has also been demonstrated in several studies [21, 23, 24, 28]. Our results are in line with findings from the Northern part of Cameroon by Ngonseau et al. [39], where peak numbers of *Bulinus truncatus* snails were reported towards the end of the dry season. Studies conducted in Nigeria showed peak abundance of *Bulinus forskalii* in the dry season and *Biomphalaria pfeifferi* at the end of the rainy season [26, 57].

Water flow rate showed a significant negative association with *Bulinus* spp. abundance, and it is well established that *Bulinus* spp prefer low flow environments [58]. Conversely, increased rainfall can provide more breeding sites for snail population through the creation of temporary and new snail habitats as there is transportation of snails by heavy rainfall [17]. In Mali, Dabo et al. [59] found a high abundance of *Bulinus truncatus* snails in slow flowing water with rocky biotope than fast flowing water, sandy and muddy substratum. Comparably, in the Tiko focus, the *Bulinus* snail species was abundant on rocky than sandy or muddy substratum habitat type. Rocky substratum habitat preference by *Bulinus* snails provide adhere to resist the water wave action thereby avoiding dislodgment [60]. The rocky biotope has other ecological advantages as it increases films of mud and fine salts that cover these rocks and favours aquatic plant growth which is used as a food source for snails [23, 61].

Our model revealed water temperature as a factor influencing the abundance of *Bulinus* sp in Tiko. Temperature has been identified as an important factor that has direct effect on freshwater snail abundance and distribution [62]. A relatively high temperature in water will lead to an increase in food availability [63], snail metabolic rate [29], and thus increasing the population size of the *Bulinus* snail by reducing the time in its developmental periods [23]. Rain may also affect cumulative impacts through sudden temperature reduction causing thermal shock in snails, reducing egg-laying success, and reducing post-rain recruitment as overall numbers will be reduced [58]. In this study, a marginal negative effect was found for medium vegetation type. *Bulinus* spp likely prefers low vegetation submerged in water and rocks to ease attachment when in water. This factor has important implications in the control of schistosomiasis. Aguiar et al. [64] reported decreased abundance of *Bulinus globosus* in sites characterized by high canopy probably related to indirect effects of canopy cover on snails through a negative impact on the ability of sunlight to reach the bottom and decrease primary production, which represents the food supply for snails. A randomized controlled trial in West Africa revealed an eightfold reduction in *Schistosoma* snail abundance following removal of submerged vegetation from water points compared to control sites. Besides disease control, this study also showed that removing vegetation offers a profitable and environmentally sound innovation for food and water access and poverty alleviation [30]. However, other studies have reported excessive vegetation cover as a factor in the spread of schistosomiasis in temperate regions [28, 65, 66]. The abundance of *Bulinus* and *Physa* was positively correlated although *Physa* was the most abundant freshwater snail species in Tiko. Some studies have shown a positive correlation between other freshwater snails (*Tarebia granifera*) and *Bulinus globosus* [67, 68] whereas a recent study showed a negative correlation, though not significant, between *B*. *globosus* and the invasive *Physa acuta* [69]. This finding has implication for further investigation as research has shown that *P*. *acuta* has higher fecundity, shorter hatching time, higher salinity tolerance, higher temperature tolerance, and tolerates fast current velocities up to 0.6 ms^-1^ compared to other pulmonates that are not found in water exceeding the velocity of 0.3 ms^-1^ [70]. Further experimental and field studies are needed to verify the use of *Physa* as a potential competitor snail for *Bulinus* spp given its numerous peculiarities over *Bulinus* spp.

## Conclusion

Schistosomiasis transmission is dependent on the presence of compatible snail intermediate hosts at water-contact sites. This study revealed the occurrence of *Bulinus* spp coexisting with other freshwater snails in intra-urban streams in Tiko with significant seasonal variation in snail abundance. The two *Bulinus* snail species including *B*. *truncatus* and *B*. *camerunensis* identified are potential intermediate hosts of *Schistosoma haematobium* in the area although none of *Bulinus* snails were found shedding schistosome cercariae. Other freshwater snails of medical and veterinary importance known to be obligate intermediate host of trematode flukes such as fascioliasis (*Lymneae*) was observed and thus present potential transmission of disease to humans and animals in contact with the water bodies. The ecological implications of the Asian species *Indoplanorbis* co-existing with *Bulinus* spp in schistosome transmission foci may be of great interest. Water temperature, flow rates and substrate type were physico-chemical variables determining the occurrence and abundance of *Bulinus* snail intermediate host in the Tiko focus. Correlation analysis suggests *Physa* snail species as potential competitor against *Bulinus-* intermediate host. Snail populations were more abundant during the dry season. This findings can inform timing of praziquantel administration in the human population as well as behavioural or snail control interventions to reduce density of freshwater snail intermediate hosts and thus control the spread of schistosomiasis at a local level.

## Supporting information

S1 File (PDF)

## Abbreviations

UGS: urogenital schistosomiasis
TDS: Total dissolve solids
EC: Electrical conductivity
T: Temperature
CDC: Cameroon Development Corporation
