# Supplementary material for: Seasonal and environmental dynamics of intra-urban freshwater habitats and their influence on the abundance of Bulinus snail host of Schistosoma haematobium in the Tiko endemic focus, Mount Cameroon region
Source: PLoS One. 2023 Oct 19;18(10):e0292943. doi: 10.1371/journal.pone.0292943 (PMC10586688; doi:10.1371/journal.pone.0292943)
Supplement: S1 File — (PDF) [file pone.0292943.s001.pdf]

Site	Name	Date	Month	North	East	Elevation	Physa
1	SN	7/12/2019	Dec	4.09036	9.34181	108	71
		12/1/2020	Jan				24
		15/02/2020	Feb				25
		18/03/2020	Mar				5
		20/04/2020	Apr				7
		22/5/2020	May				0
		26/6/2020	Jun				10
		29/07/2020	Jul				7
		28/08/2020	Aug				0
		29/09/2020	Sep				5
		27/10/2020	Oct				0
		28/11/2020	Nov				2
		13/12/2020	Dec				41
2	NL1	7/12/2019	Dec	4.08998	9.34448	69	95
		7/1/2020	Jan				55
		9/2/2020	Feb				22
		5/3/2020	Mar				6
		4/4/2020	Apr				10
		3/5/2020	May				6
		10/6/2020	Jun				12
		29/07/2020	Jul				24
		28/08/2020	Aug				42
		29/09/2020	Sep				0
		29/10/2020	Oct				5
		28/11/2020	Nov				8
		13/12/2020	Dec				21
3	NL2	17/12/2019	Dec	4.08982	9.34433	48	40
		13/1/2020	Jan				55
		15/2/2020	Feb				20
		3/11/2020	Mar				13
		8/4/2020	Apr				5
		10/5/2020	May				0
		14/6/2020	Jun				0
		27/07/2020	Jul				2
		28/08/2020	Aug				0
		29/09/2020	Sep				0
		29/10/2020	Oct				2
		28/11/2020	Nov				0
		13/12/2020	Dec				0
4	NL3	12/7/2019	Dec	4.08984	9.34472	52	93
		13/01/2020	Jan				55
		15/02/2020	Feb				40

		5/3/2020	Mar				23
		9/4/2020	Apr				13
		11/5/2020	May				21
		15/6/2020	Jun				2
		29/07/2020	Jul				1
		28/08/2020	Aug				0
		29/09/2020	Sep				0
		29/10/2020	Oct				0
		28/11/2020	Nov				0
		13/12/2020	Dec				47
5	NL4	17/12/2019	Dec	4.09012	9.34452	71	17
		16/01/2020	Jan				55
		15/02/2020	Feb				18
		11/3/2020	Mar				2
		9/4/2020	Apr				0
		11/5/2020	May				4
		16/06/2020	Jun				10
		29/07/2020	Jul				0
		28/08/2020	Aug				0
		29/09/2020	Sep				0
		29/10/2020	Oct				0
		28/11/2020	Nov				5
		13/12/2020	Dec				44
6	NL5	12/17/2019	Dec	4.08918	9.34535	68	40
		16/01/2020	Jan				39
		16/02/2020	Feb				10
		3/11/2020	Mar				13
		10/4/2020	Apr				12
		12/5/2020	May				37
		18/06/2020	Jun				14
		29/07/2020	Jul				5
		28/08/2020	Aug				0
		29/09/2020	Sep				0
		29/11/2020	Oct				0
		28/11/2020	Nov				0
		13/12/2020	Dec				14
7	NL6	5/12/2019	Dec	4.08886	9.3455	69	0
		16/01/2020	Jan				435
		16/02/2020	Feb				223
		4/3/2020	Mar				7
		2/4/2020	Apr				6
		13/05/2020	May				4
		19/06/2020	Jun				10

		29/07/2020	Jul				13
		28/08/2020	Aug				0
		29/09/2020	Sep				0
		29/10/2020	Oct				0
		28/11/2020	Nov				0
		13/12/2020	Dec				22
8	NL7	5/12/2019	Dec	4.08868	9.34546	75	0
		16/01/2020	Jan				22
		16/02/2020	Feb				21
		4/3/2020	Mar				0
		2/4/2020	Apr				22
		13/05/2020	May				0
		19/06/2020	Jun				0
		29/07/2020	Jul				13
		28/08/2020	Aug				0
		29/09/2020	Sep				4
		29/10/2020	Oct				0
		28/11/2020	Nov				0
		13/12/2020	Dec				78
9	NC	6/12/2019	Dec	4.07952	9.35724	61	5
		16/01/2020	Jan				2
		16/2/2020	Feb				38
		4/3/2020	Mar				0
		2/4/2020	Apr				16
		5/19/2020	May				4
		19/06/2020	Jun				0
		30/07/2020	Jul				10
		29/08/2020	Aug				0
		30/09/2020	Sep				0
		29/10/2020	Oct				0
		28/11/2020	Nov				0
		13/12/2020	Dec				41
10	NH	6/12/2019	Dec	4.08315	9.36255	54	0
		16/01/2020	Jan				0
		2/11/2020	Feb				2
		4/3/2020	Mar				0
		4/3/2020	Apr				4
		5/19/2020	May				0
		19/06/2020	Jun				0
		29/07/2020	Jul				0
		29/08/2020	Aug				22
		29/09/2020	Sep				0
		29/10/2020	Oct				0

		28/11/2020	Nov				0
		13/12/2020	Dec				0
11	NWT1	6/12/2019	Dec	4.06821	9.36538	18	0
		16/01/2021	Jan				7
		2/11/2020	Feb				14
		4/3/2020	Mar				0
		4/3/2020	Apr				0
		5/26/2020	May				0
		19/06/2020	Jun				0
		30/07/2020	Jul				4
		29/08/2020	Aug				21
		29/09/2020	Sep				0
		29/10/2020	Oct				0
		28/11/2020	Nov				0
		13/12/2020	Dec				0
12	NWT2	6/12/2019	Dec	4.0673	9.36588	11	0
		16/01/2020	Jan				0
		2/11/2020	Feb				12
		4/3/2020	Mar				8
		4/4/2020	Apr				2
		5/26/2020	May				5
		19/06/2020	Jun				30
		30/07/2020	Jul				21
		29/08/2020	Aug				18
		29/09/2020	Sep				10
		29/10/2020	Oct				5
		28/11/2020	Nov				0
		13/12/2020	Dec				14

Sites purposively selected during human contact activities and included

SN = SNEC

NL = Ndongo_Likomba

NC = Ndongo_Costene

NH = Ndongo_Holforth

NWT = Ndongo_Water tank

Bulinus	Melanoide	Lymneae	Potadoma	Biomphalaria	SOIL_TYPE	VEGETATION	WATER_DEPTH(m)
2	12	5	3	0	sandy	medium	0.23
0	3	0	0	0	sandy	medium	0.25
0	0	0	0	0	sandy	medium	0.21
0	12	2	0	0	sandy	medium	0.21
5	39	2	0	0	sandy	medium	0.33
0	6	0	0	0	sandy	medium	0.45
0	2	11	0	0	sandy	medium	0.33
0	3	20	0	0	sandy	medium	0.23
0	0	0	0	0	sandy	medium	0.21
0	0	0	0	0	sandy	medium	0.18
0	0	0	0	0	sandy	medium	0.15
0	14	0	0	0	sandy	medium	0.02
0	11	0	0	0	sandy	medium	0.18
0	0	1	0	0	sandy	Low	0.25
0	0	3	0	0	sandy	Low	0.25
0	0	0	0	0	sandy	Low	0.24
6	10	0	0	0	sandy	Low	0.23
10	10	0	0	0	sandy	Low	0.23
6	8	0	0	0	sandy	Low	0.23
9	16	0	0	0	sandy	Low	0.26
0	3	0	0	0	sandy	Low	0.55
3	5	2	0	0	sandy	Low	0.5
0	2	0	0	0	sandy	Low	0.57
0	0	0	0	0	sandy	Low	0.65
2	0	0	0	0	sandy	Low	0.26
6	4	0	0	0	sandy	Low	0.26
36	0	8	0	0	Rocky	Low	0.37
40	0	5	0	0	Rocky	Low	0.4
10	0	0	0	0	Rocky	Low	0.4
0	0	0	0	0	Rocky	Low	0.38
0	12	0	0	0	Rocky	Low	0.41
0	2	0	0	0	Rocky	Low	0.5
0	0	0	0	0	Rocky	Low	0.56
0	7	0	0	0	Rocky	Low	0.59
0	0	0	0	0	Rocky	Low	0.55
0	9	0	0	0	Rocky	Low	0.57
3	0	0	0	0	Rocky	Low	0.66
0	0	0	0	0	Rocky	Low	0.27
0	0	0	0	0	Rocky	Low	0.39
12	0	0	0	0	sandy	Low	0.29
6	0	0	0	0	sandy	Low	0.3
36	0	8	0	0	sandy	Low	0.37

2	0	0	0	0	sandy	Low	0.3
0	0	0	0	0	sandy	Low	0.38
0	0	0	0	0	sandy	Low	0.38
0	8	0	0	0	sandy	Low	0.45
0	39	1	0	0	sandy	Low	0.74
0	4	0	0	0	sandy	Low	0.77
0	0	0	0	0	sandy	Low	0.57
0	0	0	0	0	sandy	Low	0.55
0	0	0	0	0	sandy	Low	0.37
16	0	2	0	0	sandy	Low	0.37
6	0	0	0	0	sandy	Low	0.43
10	0	0	0	0	sandy	Low	0.38
5	0	0	0	0	sandy	Low	0.44
0	8	0	0	0	sandy	Low	0.45
0	16	0	0	0	sandy	Low	0.45
0	0	0	0	0	sandy	Low	0.36
2	0	0	0	0	sandy	Low	0.4
0	9	0	0	0	sandy	Low	0.5
0	22	0	0	0	sandy	Low	0.61
0	0	0	0	0	sandy	Low	0.57
0	0	0	0	0	sandy	Low	0.55
5	0	1	0	0	sandy	Low	0.27
22	0	0	0	0	sandy	Low	0.29
36	0	8	0	0	ROCKY	LOW	0.37
16	0	3	0	0	ROCKY	LOW	0.37
2	0	0	0	0	ROCKY	LOW	0.4
0	0	0	0	0	ROCKY	LOW	0.38
4	0	0	0	0	ROCKY	LOW	0.28
0	0	0	0	0	ROCKY	LOW	0.29
10	0	0	0	0	ROCKY	LOW	0.3
0	69	0	0	0	Rocky	Low	0.54
0	70	0	0	0	Rocky	Low	0.59
0	0	0	0	0	Rocky	Low	0.57
0	0	0	0	0	Rocky	Low	0.57
0	0	0	0	0	Rocky	Low	0.27
55	5	0	0	0	Rocky	Low	0.38
0	11	0	0	0	SANDY	LOW	0.49
212	0	0	0	0	SANDY	LOW	0.22
140	0	0	0	0	SANDY	LOW	0.23
0	38	0	0	0	SANDY	LOW	0.28
0	10	2	0	0	SANDY	LOW	0.46
0	5	5	0	0	SANDY	LOW	0.42
0	5	1	0	0	SANDY	LOW	0.31

0	2	5	0	0	sandy	Low	0.42
0	0	0	0	0	sandy	Low	0.41
0	0	0	0	0	Rocky	Low	0.57
0	0	0	0	0	Rocky	Low	0.57
0	0	0	0	0	Rocky	Low	0.27
19	0	0	0	0	Rocky	Low	0.38
0	0	0	0	0	SANDY	LOW	0.45
3	0	0	0	0	SANDY	LOW	0.44
0	4	1	0	0	SANDY	LOW	0.42
0	0	0	0	0	SANDY	LOW	0.45
3	0	0	0	0	SANDY	LOW	0.44
0	0	0	0	0	SANDY	LOW	0.45
0	0	0	0	0	SANDY	LOW	0.44
0	2	5	0	0	sandy	Low	0.42
0	0	0	0	0	sandy	Low	0.12
0	0	0	0	0	Rocky	Low	0.65
0	0	0	0	0	Rocky	Low	0.57
0	0	0	0	0	Rocky	Low	0.27
40	0	0	0	0	Rocky	Low	0.38
1	5	0	0	0	MUDDY	LOW	0.4
0	90	1	0	0	MUDDY	LOW	0.3
0	5	5	0	0	MUDDY	LOW	0.38
0	15	0	0	0	MUDDY	LOW	0.4
3	16	0	0	0	MUDDY	LOW	0.4
0	13	0	0	0	MUDDY	LOW	0.39
0	0	0	0	0	MUDDY	LOW	0.48
0	8	4	0	0	MUDDY	LOW	0.32
0	25	0	0	0	MUDDY	LOW	0.36
0	0	3	0	0	MUDDY	LOW	0.3
0	0	0	0	0	MUDDY	LOW	0.3
0	50	0	0	0	MUDDY	LOW	0.4
12	8	7	0	0	MUDDY	LOW	0.38
0	30	1	0	0	MUDDY	MEDIUM	0.3
0	14	1	0	0	MUDDY	MEDIUM	0.3
0	90	1	0	0	MUDDY	MEDIUM	0.3
0	0	0	0	0	MUDDY	LOW	0.4
4	7	0	0	0	MUDDY	MEDIUM	0.49
0	22	0	0	0	MUDDY	MEDIUM	0.33
0	0	0	0	0	MUDDY	MEDIUM	0.5
0	0	0	0	0	MUDDY	MEDIUM	0.49
0	4	0	0	0	MUDDY	LOW	0.43
0	0	0	0	0	MUDDY	LOW	0.4
0	0	0	0	0	MUDDY	LOW	0.4

0	0	0	0	0	MUDDY	LOW	0.13
0	0	0	0	0	MUDDY	LOW	0.15
0	0	0	0	0	MUDDY	MEDIUM	0.44
0	0	0	0	0	MUDDY	MEDIUM	0.45
2	3	1	0	2	MUDDY	MEDIUM	0.45
0	0	0	0	0	MUDDY	MEDIUM	0.4
0	5	0	0	0	MUDDY	MEDIUM	0.49
0	0	0	0	0	MUDDY	MEDIUM	0.47
0	0	0	0	0	MUDDY	MEDIUM	0.5
0	0	0	0	0	MUDDY	medium	0.53
0	0	0	0	0	MUDDY	medium	0.45
0	0	0	0	0	MUDDY	medium	0.45
0	0	0	0	0	MUDDY	medium	0.45
0	0	0	0	0	MUDDY	medium	0.47
0	0	0	0	0	MUDDY	medium	0.47
0	0	0	0	0	ROCKY	MEDIUM	0.45
0	5		0	0	ROCKY	MEDIUM	0.4
6	5	1	0	0	ROCKY	MEDIUM	0.4
6	5	1	0	0	ROCKY	MEDIUM	0.45
4	2	1	0	0	ROCKY	MEDIUM	0.45
0	0	0	0	1	ROCKY	MEDIUM	0.43
5	0	0	0	0	Rocky	medium	0.45
2	0	0	0	0	Rocky	medium	0.45
0	5	0	0	0	Rocky	medium	0.45
0	0	0	0	0	Rocky	medium	0.25
3	0	0	0	0	Rocky	medium	0.25
1	0	0	0	0	Rocky	medium	0.35
26	0	5	0	1	Rocky	medium	0.38

d for snail sampling

WATER_WIDTH(m)	FLOW_VEL(m/s)	FLOW_RATE(m3/s)	pH	TDS	EC	TEMP
0.17	0.67	0.03	8.58	103	203	30
0.18	0.67	0.03	8.63	97.2	189	29.8
0.18	0.67	0.03	8.63	100.2	180	29.8
0.14	0.67	0.02	8.31	115	220	28.5
0.17	0.55	0.03	8.21	112	227	28
10.2	0.38	1.74	8.34	111	221	28.1
0.19	0.15	0.009	5.8	30.8	177	27.8
0.19	0.09	0.004	5.8	33.8	166	27.5
0.11	0.15	0.003	6.5	80.9	161	26.4
0.09	0.07	0.001	7	0.08	150	26.4
0.08	0.05	0.0006	8	0.08	140	26.2
0.04	0.24	0.0002	7	44	217	27.9
0.9	0.24	0.04	6.8	33	150	26.4
10.9	0.24	0.6	8.36	112	217	30.2
9.9	0.26	0.64	8.3	109	220	29.8
9.9	0.26	0.64	7.8	109	210	29
10.55	0.26	0.6	8.09	110	223	28.8
9.5	0.24	0.52	8	120	222	27.9
10.55	0.26	0.63	8.09	110	223	28.8
10.5	0.3	0.8	8.09	88	220	27.5
9.8	0.36	1.9	6.7	77	154	26.7
10	0.37	1.8	6	84.2	171	26.5
12	1.25	8.5	8	45	148	27.2
11	1.12	10.2	8.1	48	154	27.4
11	1.25	3.6	7.2	0.11	228	27.1
11	1.11	3.2	6.9	0.11	224	28.2
10.1	0.38	1.4	8.37	112	229	31.2
10.1	0.29	1.1	8.1	111	222	30.5
10.1	0.29	1.1	8.7	115	219	30.6
10	0.42	1.6	8.4	113	220	28.9
9.7	0.48	1.91	8.24	116	221	27.1
10.1	0.55	2.8	7	101	217	27.5
10.1	0.55	2.8	7	90	198	27
11.6	0.91	6.2	6.8	77.3	154	26.8
11.2	0.91	5.6	6.6	80.1	160	26.7
12	1.25	8.5	8	89	148	27.2
11	1.12	8.1	8.1	55	160	26.3
13	0.37	1.3	7.4	88	234	27.6
12	0.11	0.5	6.1	80	144	27.5
8.6	0.2	0.5	8.34	107	219	32
8.6	0.2	0.5	7	110	221	30.2
10.1	0.38	1.42	8.37	112	229	31.2

8.45	0.21	0.5	8.13	113	225	28.3
10	0.42	1.59	8.4	113	220	28.9
9.65	0.43	1.58	8.37	115	223	28.5
9.5	0.53	2.27	8.32	117	226	29
11.8	0.91	7.9	7	78.8	158	26.4
11.7	0.91	8.2	7.2	77.6	159	26.6
12	1.25	8.5	8	80	160	26.8
11	1.12	6.8	7.2	56	160	26.3
11	0.91	3.7	6.7	102	189	27
11	0.67	2.7	7.1	48	155	26.7
9.7	0.5	2.2	8.4	112	229	30.5
9.4	0.53	1.93	8.4	114	219	30.8
7.7	0.36	1.22	8.7	113	219	29
9.5	0.53	2.3	8.32	117	226	29
7.5	0.5	1.69	8.38	113	227	27.8
5.9	0.53	1.13	8.33	112	225	27.6
6.7	0.48	1.29	8.4	117	225	28.2
11.8	0.45	2.7	6.9	78.3	168	26.5
11.7	0.4	2.8	7	78.3	170	26.7
12	1.25	8.5	6.8	89	158	26.8
11	0.4	2.4	8	79	189	26.8
12	0.4	1.3	7.5	102	145	26.8
11	0.37	1.2	7.4	79	180	26.9
10.1	0.38	1.42	8.37	112	229	31.2
6	0.42	0.93	8.66	111	223	30.5
6.7	0.48	1.29	8.4	117	225	28.2
10	0.42	1.59	8.4	113	220	28.9
8.7	0.25	0.61	8.31	115	229	27.8
11.75	0.34	1.16	8.37	115	228	27.5
12	0.31	1.12	8.25	113	229	28.3
12	0.75	4.86	7.5	83.6	166	26.7
12	0.75	5.31	7	80.6	170	26.8
12	1.5	10.26	6	80	148	27.2
11	1.3	8.6	8	84	188	27.4
10	1.25	3.3	8	112	144	26.5
10	0.42	1.59	8.4	118	200	27.6
12.35	0.53	3.21	8.18	118	229	27.1
10.8	0.196	0.47	8.05	114	229	29.7
10.5	0.29	0.7	7.94	117	232	29.3
10.6	0.36	0.99	8.14	117	228	27.9
12.2	0.4	2.24	8.1	117	231	30.23
11.1	0.5	2.2	8	118	234	29.5
12	0.5	1.8	8	111	205	29.5

11	1	4.6	6	81.1	161	26.9
11	0.9	4	6	80.2	171	26.8
12	1.5	10.26	6	80	148	27
11	1.3	8.6	8	102	145	26.8
10	1.25	3.3	8	97	147	26.7
10	0.42	1.59	8.4	118	200	27.6
12.85	0.5	2.89	8.09	119	230	28.6
13	0.45	2.57	7.42	113	227	30.6
5.4	0.43	0.98	7.43	112	214	29.5
7	0.53	1.67	8.15	115	229	27.4
13	0.45	2.57	7.42	113	227	30.6
12.85	0.5	2.89	8.09	119	230	28.6
12	0.5	2.9	8	122	221	28.6
11	1	4.6	6	81.1	161	26.9
41	1	4.9	7	80.1	165	27.1
12	1.5	11.7	6	80	148	27.2
11	1.3	8.6	8	102	145	26.8
10	1.25	3.3	8	97	147	26.7
10	0.42	1.59	8.4	118	200	27.6
8.2	0.42	1.4	8	115	210	29.8
9.3	0.36	1	7.89	111	211	30.4
4.1	0.4	0.62	8.14	116	220	29.7
8.25	0.38	1.25	8.13	114	218	27.6
8.1	0.32	1.04	8.1	112	209	29.9
4.1	0.42	0.67	8.05	117	226	27.9
7.45	0.53	1.89	8.1	118	225	28.8
120	0.17	6.5	7.5	102	210	26.6
9	0.5	0.18	6	91.4	178	26.6
17	0.9	4.6	8	80	233	28.3
18	0.9	4.9	8	83	200	26.7
17	0.4	2.7	8	112	168	26.7
4.1	0.4	0.62	8.14	116	220	29.7
9.3	0.36	1	7.5	120	209	30.5
9.8	0.34	1	7.6	112	209	29.8
9.3	0.36	1	7.89	111	211	30.4
8.25	0.38	1.25	8.13	114	218	27.6
7.4	0.5	1.81	7.38	115	221	30
9.4	0.4	1.24	8.08	114	222	28.1
10	0.9	4.5	7.5	75.8	154	26.5
10	0.8	3.92	7.7	77.9	155	25.8
8	0.4	1.6	6.1	112	228	27.6
2	0.8	0.6	8	0.18	288	28.4
2	0.8	0.6	8	0.14	269	29.6

17	0.2	0.44	7	0.11	280	27.7
17	0.2	0.51	7	0.13	260	29.5
4.5	0.55	1.1	8	118	220	28.5
4.5	0.45	0.91	7.89	122	224	30
4.6	0.48	0.99	8.12	114	219	30.5
4	0.38	1.9	8.13	114	218	27.6
7.4	0.5	1.81	7.38	115	221	30
4.5	0.53	1.12	8.2	118	222	28.9
10	0.9	4.5	7.5	75.8	154	26.5
6	1	3.8	7.9	88.3	182	26.5
5	0.5	1.2	6.7	99	213	27.2
4	0.5	0.9	8	0.13	306	29.3
4	0.5	0.9	8	0.13	306	29.3
4	0.5	0.9	8	0.14	300	28.9
4	0.5	0.9	8	0.13	289	29.3
4	0.48	0.98	8	115	220	30.5
5.1	0.48	0.98	8	120	214	29.8
5.1	0.48	0.98	7.99	113	217	30.6
5.1	0.5	1.1	7.5	117	221	29.5
5.1	0.5	1.1	7.5	220	222	28.9
5.3	0.48	1.09	8.12	115	226	29
5.5	0.47	1.1	7.5	90	180	26.5
5	0.47	1.07	7.8	88.4	177	26.8
4	0.5	0.9	8	110	216	26.7
6	0.45	0.68	6	89	272	29.9
6	0.45	0.68	6	89	245	28.4
5	0.45	0.78	7.2	97	277	27.9
2	0.52	0.39	7	110	305	28.8

SALINITY
0.15
0.12
0.12
0.09
0.09
0.1
0.07
0.07
0.07
0.23
0.25
0.08
0.23
0.11
0.11
0.11
0.08
0.07
0.08
0.07
0.06
0.08
0.07
0.07
0.08
0.08
0.10
0.09
0.09
0.09
0.08
0.07
0.07
0.07
0.07
0.07
0.08
0.1
0.12
0.10
0.09
0.10

0.09
0.09
0.09
0.09
0.06
0.06
0.06
0.08
0.08
0.06

0.10
0.11
0.09
0.09
0.1
0.09
0.09
0.05
0.06
0.05
0.06
0.06
0.1

0.10
0.13
0.09
0.09
0.09
0.09
0.1
0.06
0.06
0.07
0.08
0.06
0.09

0.09
0.11
0.09
0.09
0.10
0.10
0.10

0.05
0.07
0.06
0.07
0.06
0.09

0.08
0.08
0.09
0.09
0.08
0.08
0.08
0.05
0.06
0.06
0.07
0.06
0.09

0.10
0.10
0.11
0.09
0.10
0.09
0.1
0.07
0.12
0.07
0.06
0.06
0.11

0.09
0.10
0.10
0.09
0.10
0.10
0.06
0.06
0.06
0.14
0.11

0.14
0.11

0.10
0.09
0.10
0.09
0.10
0.10
0.06
0.07
0.06
0.09
0.09
0.09
0.09

0.10
0.09
0.10
0.09
0.10
0.09
0.07
0.07
0.07
0.09
0.07
0.06
0.06
